# Characteristics of lymph node metastasis and short-term outcome of esophageal squamous-cell carcinoma undergoing minimally invasive esophagectomy: a prospective cross-sectional study (with video)

**DOI:** 10.1097/MS9.0000000000002500

**Published:** 2024-09-04

**Authors:** Duy Duc Nguyen, Binh Van Pham, Manh Dai Tran, Thanh Duy Nguyen, An Duc Thai, Ky Van Le, Vu Van Kim, Hung Xuan Nguyen

**Affiliations:** a Hanoi Medical University; b Department of Abdominal Surgery 1, Vietnam National Cancer Hospital; c Pathology and Molecular Biology Center, Vietnam National Cancer Hospital; d Hong Ngoc Hospital, Hanoi, Vietnam

**Keywords:** esophageal squamous-cell carcinoma, minimally invasive esophagectomy, extended two-field lymph node dissection

## Abstract

**Introduction:**

Surgery for esophageal squamous-cell carcinoma (ESCC) presents many potential challenges owing to malignant lymph node metastasis, complex procedures and severe postoperative complications. The appropriate lymphadenectomy for ESCC remains controversial. This study aims to evaluate the characteristics of lymph node metastasis and postoperative complications in patients with ESCC undergoing minimally invasive esophagectomy and extended two-field lymph node dissection.

**Patients and methods:**

This prospective, single-center, cross-sectional study was conducted from October 2022 to May 2024. All patients with ESCC who underwent minimally invasive esophagectomy and extended two-field lymph node dissection were selected for this study. Postoperative lymph nodes were divided into upper thoracic, middle thoracic, lower thoracic and abdominal lymph node groups.

**Results:**

Seventy-four patients with ESCC, including 49 patients who underwent upfront surgery and 25 patients who received preoperative chemoradiotherapy, were selected. The rate of lymph node metastasis in all patients was 39.2%, with 13.6% of patients having upper thoracic metastasis. The factors affecting the rate of lymph node metastasis included preoperative chemoradiotherapy, tumor stage, poor differentiation, lymphovascular/perineural invasion, and tumor size greater than 2 cm, all of which were significantly different (*P*<0.05). Common postoperative complications included pneumonia (25.7%), recurrent laryngeal nerve (RLN) palsy (10.8%) and anastomotic leak (4.1%). There were no cases required conversion to open surgery, nor any deaths within 90 days postoperatively.

**Conclusion:**

Lymph node metastasis in esophageal squamous-cell carcinoma has a high incidence, occurs in the early stages, and is widely distributed in all regions of the mediastinum and abdomen. Minimally invasive esophagectomy and extended two-field lymph node dissection are feasible and safe, with low complication rates.

## Introduction

Highlights

- Lymph node metastasis in esophageal squamous-cell carcinoma has a high incidence, occurs in the early stages, and is widely distributed.
- Minimally invasive esophagectomy and extended two-field lymph node dissection are feasible and safe, with low complication rates.
- Factors influencing the characteristics of lymph node metastasis include preoperative chemoradiotherapy, tumor stage, poor differentiation, lymphovascular/perineural invasion, and tumor size greater than 2 cm.

Esophageal cancer is one of the diseases with a poor prognosis among gastrointestinal cancers with a 5-year survival time of less than 20%^1^. According to GLOBOCAN 2022^2^, esophageal cancer ranks 11th in the number of new cases but ranks 7th in the number of deaths. Current esophageal cancer treatment involves a multi-modality approach, in which esophagectomy and lymph node dissection are radical treatment methods for early or locally advanced stages without distant metastases.

Lymph node metastasis in ESCC is especially complex and widespread. Radical lymph node dissection is an important factor that increases survival time and reduces the rates of local recurrence and distant metastasis. However, this is a complex technique with the risk of serious complications for the patient, requiring qualified surgeons and anesthesiologists, as well as modern facilities. The Japanese Esophageal Society recommends mediastinal extended lymphadenectomy as a mandatory for the treatment of thoracic esophageal cancer^3^. However, performing upper mediastinal lymphadenectomy, including the 106Rec group located close to the recurrent laryngeal nerve, can lead to serious postoperative respiratory and cardiovascular complications. Therefore, this is the most challenging aspect of esophageal cancer surgery^4^.

This study aims to evaluate the characteristics of lymph node metastasis and postoperative complications in patients with ESCC undergoing minimally invasive esophagectomy and extended two-field lymph node dissection.

## Patients and methods

### Data collection

This is a prospective cross-sectional study involving 74 selected patients, all diagnosed with thoracic ESCC. The indication for upfront surgery or preoperative chemoradiotherapy (CRT) followed the National Comprehensive Cancer Network recommendations^5^. All patients underwent three-dimensional thoracolaparoscopic esophagectomy and extended two-field lymph node dissection (including upper thoracic lymph nodes according to Matsuda’s classification^6^). This study was conducted from October 2022 to May 2024.

### Surgical procedure

Thoracic Phase (video, Supplemental Digital Content 2, http://links.lww.com/MS9/A594): We used two positions: semi-prone or a hybrid of semi-prone and left lateral. Five trocars were placed in both positions. Lymph node dissection was performed according to the recommendations of the Japanese Esophageal Society^3^. The middle and lower thoracic lymph nodes were dissected first. The right recurrent laryngeal nerve (RLN) lymph nodes were dissected later by opening the superior mediastinal pleura along the vagus nerve and finding the root of the RLN. Subsequently, the left RLN lymph nodes were removed. The trachea was retracted to the right, and the fat layer containing the lymph nodes was removed from the starting position under the aortic arch to the cervical paraesophageal lymph nodes. Dissection of this lymph node group was performed in the semi-prone or left lateral position (video, Supplemental Digital Content 2, http://links.lww.com/MS9/A594).

*Abdominal phase*: The abdominal phase was also performed laparoscopically, including lymph node dissection of the group around the celiac artery, lesser curvature, and bilateral diaphragmatic crus. Reconstruction was performed by using a gastric tube. Anastomosis was completed at the neck using either a circular stapler or the hand-sewn technique.

Postoperatively, the patient was immediately extubated and placed in the intensive care unit. Postoperative complications were monitored, diagnosed, and classified according to the Japan Clinical Oncology Group postoperative complication criteria, based on the extended Clavien–Dindo classification^7^.

The postoperative lymph node groups were divided as follows: upper thoracic (105, 106RecR, 106RecL, 106tbL), middle thoracic (107, 108, 109 bilaterally), lower thoracic (110, 111, 112Pul, 112Ao), and abdominal (1, 2, 3a, 7, 8, 9, 11p, 19, 20). Postoperative pathology was reported by a single experienced pathologist.

### Statistical analysis

Data collection and analysis were conducted using the SPSS software (version 20, SPSS Inc.). Risk factors for lymph node metastasis were assessed using the χ^2^ or Fisher’s exact test. A *p* value less than 0.05 with a 95% CI was considered statistically significant.

This prospective, cross-sectional study has been reported in line with the Strengthening the Reporting of Cohort, Cross-sectional and case-control studies in Surgery (STROCSS, Supplemental Digital Content 1, http://links.lww.com/MS9/A593) guideline^8^.

## Results

**Figure 1:**
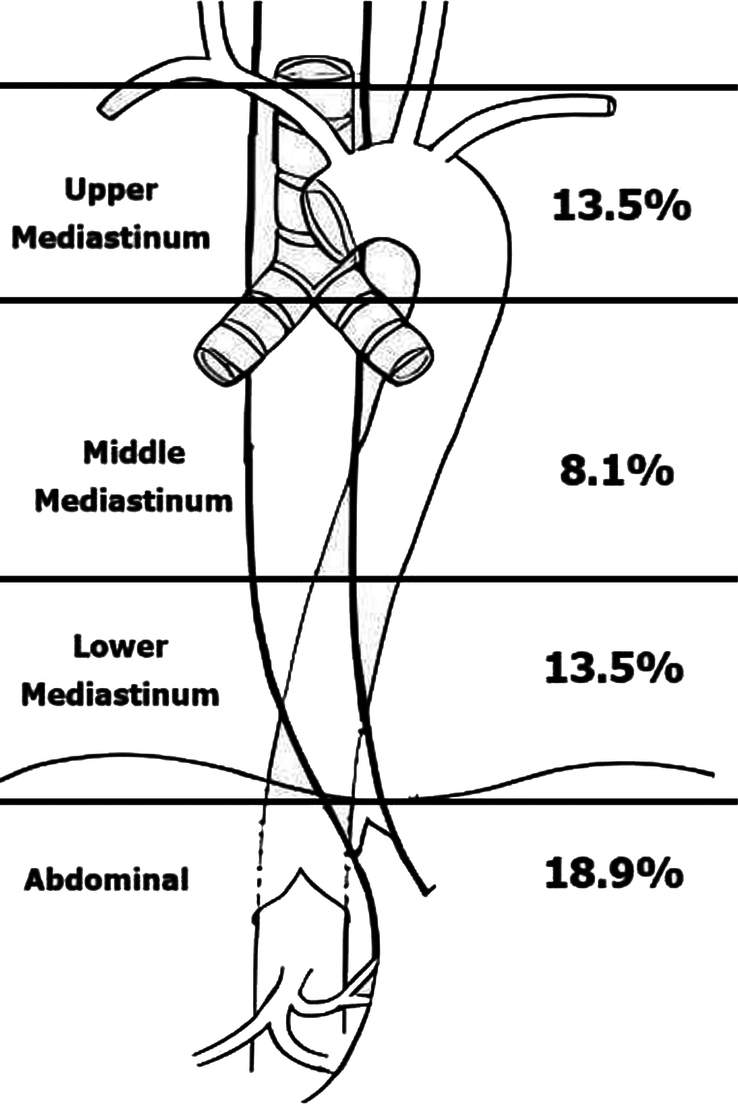
Rate of lymph node metastasis divided by lymph node group.

Among the 74 patients who underwent minimally invasive esophagectomy and extended two-field lymph node dissection, we recorded an overall lymph node metastasis rate of 39.2%. Specifically, metastasis to the upper thoracic group was 13.5%, and abdominal lymph node metastasis was 18.9% (as seen in Fig. 1).

As seen in Table 1, lymph node metastasis in EC occurs early, with a rate of 26.7% at stage T1. Significant factors influencing the characteristics of lymph node metastasis were recorded, including preoperative chemoradiotherapy (*P*=0.03), tumor stage (*P*=0.01), poor differentiation (*P*=0.01), lymphovascular/perineural invasion (*P*=0.04), and tumor size greater than 2 cm (*P*=0.03).

**Table 1:** Analyze the relationship between related factors and lymph node metastasis characteristics

Factors	LN-positive(n = 29)	LN-negative (n = 45)	*p*
Age (year) mean ± SD	56.3 ± 7.5	56.6 ± 6.5	0.86
Chemoradiotherapy, *n* (%)			
No	15 (30.6)	34 (69.4)	*P* = 0.03 RR =2.9, 95% CI: 1.1–7.8
Yes	14 (56)	11 (44)	
Tumor location, *n* (%)			
Middle thoracic	9 (29)	22 (71)	*P* = 0.13
Lower thoracic	20 (46.5)	23 (53.5)	
T stage, *n* (%)			
T1	12 (26.7)	33 (73.3)	*P* = 0.01
T2	6 (75)	2 (25)	
T3	7 (66.7)	3 (33.3)	
No residual tumor post-CRT	4 (36.4)	7 (63.6)	
Differentiation, *n* (%)			
Moderate	14 (35)	26 (65)	*P* = 0.01 RR =7.4, 95% CI: 1.4–39.9
Poor	8 (80)	2 (20)	
Lymphovascular/perineural invasion, *n* (%)			
Yes	7 (70)	3 (30)	*P* = 0.04 RR =4.5, 95% CI: 1.04–18.9
No	22 (34.4)	42 (65.6)	
Tumor length (cm), *n* (%)			
≤2 cm	12 (28.6)	30 (71.4)	*P* = 0.03 RR =2.8, 95% CI: 1.1–7.4
>2 cm	17 (53.1)	15 (46.9)	

CRT, chemoradiotherapy; RR, risk ratio.

For intraoperative complications, there were three cases of pleural adhesions and one case of tracheal injury, with no cases requiring conversion to open surgery. The most common postoperative complications were pneumonia (25.9%) and recurrent laryngeal nerve palsy (RLNP) (10.8%). It was noted that 9.5% of cases had severe complications classified as Clavien–Dindo grade greater than or equal to 3. The average number of retrieved recurrent laryngeal nerve lymph nodes was 11.4 ± 8.3, and the total number of retrieved lymph nodes was 38.5 ± 14.8, as demonstrated in Table 2.

**Table 2:** Patient demographics, surgical outcomes and postoperative complications

Characteristics	Result [*n* (%)]
Age (mean ± SD)	56.5 ± 6.8 (min 37, max 69)
Sex	
Male	73
Female	1
Tumor location	
Middle thoracic	31 (41.9)
Lower thoracic	43 (58.1)
CRT	
No	49 (66.2)
Yes	25 (33.8)
pN stage	
N0	45 (60.8)
N1	28 (37.8)
N2	1 (1.4)
Operation time (min) (mean ± SD)	319.8 ± 64.1 (min 210, max 500)
Intraoperative blood loss (ml) (mean ± SD)	149.9 ± 78.8 (min 210, max 380)
Intraoperative complication	
Pleural adhesions	3 (4.1)
Emphysema	2 (2.7)
Tracheal trauma	1 (1.4)
Conversion to thoracotomy	0
Mean postoperative ICU stay (days)	4.6 ± 2.1 (min 2, max 13)
Mean postoperative hospital stay (days)	12.3 ± 4.6 (min 7, max 30)
Surgical complications	
Left recurrent laryngeal nerve palsy	8 (10.8)
Anastomotic leakage	3 (4.1)
Postoperative bleeding	2 (2.7)
Chylothorax	0
Non-surgical complications	
Pneumonia	19 (25.7)
Arrhythmia	3 (4.1)
Mortality within 90 days	0
Severe complications (CD grade ≥ IIIa)	
Yes	7 (9.5)
No	67 (90.5)
No. retrieved lymph nodes	
Recurrent laryngeal nerve	11.4 ± 8.3 (min 2, max 55)
Thoracic	25.6 ±11.8 (min 9, max 74)
Abdominal	10.9 ± 5.4 (min 2, max 25)
Total	38.5 ± 14.8 (min 17, max 83)

CD, Clavien–Dindo; CRT, chemoradiotherapy; max, maximum; min, minimum.

## Discussion

In our study, lymph node metastasis in esophageal squamous-cell carcinoma has the following characteristics:

### First of all, lymph node metastasis in esophageal cancer occurs from early stages

Our study showed that lymph node metastasis rates for T1, T2 and T3 was 26.7%, 75%, and 66.7%, respectively. Thus, as the primary tumor progressed to later stages, the rate of lymph node metastasis increased significantly (*P* = 0.01). This is because the esophagus has a complex lymphatic drainage system. Except for the epithelium and lamina propria, there is a complicated lymphatic network from the muscularis mucosae outward. There are many lymphatic drainage pathways between the submucosa and extramural lymphatic system, including the thoracic duct and lymph nodes^9^. Consequently, in clinical practice, it is necessary for surgeons to perform lymph node dissection according to the guidelines without limitations, even in early-stage EC.

Lymph node metastasis occurs widely in the mediastinal and abdominal regions in esophageal cancer (EC). This widespread metastasis is explained by the circulation of lymphatics from the inner layer of the esophagus in various directions: outward, upward, or downward. Lymph flow in the upper two-thirds of the esophagus tends to move upward, whereas in the lower third, it tends to move downward^9^. However, all lymphatics are interconnected. Therefore, lymphatic fluid from any part of the esophagus can spread, facilitating extensive metastasis in EC.

This study noted that the factors affecting the rate of lymph node metastasis included preoperative CRT (*P*=0.03), tumor stage (*P*=0.01), poor differentiation (*P*=0.01), lymphovascular/perineural invasion (*P*=0.04), and tumor size greater than 2 cm (*P*=0.03). Tumor size is a predictive factor for the rate of lymph node metastasis^10^. In addition, patients in the CRT group had a higher rate of lymph node metastasis, which may be because the CRT group had more advanced stages and one of the indications for CRT was preoperative lymph node metastasis. Currently, there is still debate regarding whether lymph node dissection should be extended to patients who have undergone preoperative CRT^11^. This study showed that the incidence of lymph node metastasis was up to 56% in this group. Therefore, lymph node dissection was necessary.

We noted a high rate of upper thoracic lymph node metastasis at all stages, similar to reports from Eastern countries^9^. Therefore, extended two-field lymphadenectomy should be prescribed, as per the Japanese Esophageal Society 2022 guideline, which mandates the dissection of this group for all thoracic esophageal cancers^3^. However, RLN lymph node groups pose significant challenges for surgeons because of the high risk of postoperative complications related to the respiratory and cardiovascular systems. In this study, the RLNP rate was 10.8%. Compared with other studies, this rate varied widely, from 0 to 60%^4,12,13^. All RLNP cases occurred on the left side and in patients who were positioned semi-prone. This is because the nerve is obscured by the trachea in this position. Moreover, the knife hand is oriented perpendicular to the nerve, and there is conflict between the main and assistant surgeons, leading to increased tension and potential damage to the nerve. To address this issue, we made improvements and performed lymph node dissection in the left lateral position, which helped to overcome the aforementioned drawbacks and reduce the incidence of palsy to 0%.

Although lymph node dissection was performed in the upper thoracic group, other postoperative complications in our study had a low rate of severe complications (Clavien–Dindo ≥ 3), occurring in 9.5%, and the rates of pneumonia and anastomotic leak were 25.7% and 4.1%, respectively, and there were no cases of death within 90 days postoperatively. Therefore, we believe that extending the lymph node dissection field, including the upper thoracic group, is safe, feasible, and necessary in cases of thoracic ESCC.

### Limitations

Our study did not divide each lymph node in detail according to the Japanese Esophageal Society guidelines; instead, we dissected them into upper, middle, lower thoracic, and abdominal groups due to financial obstacles.

### Implications

Lymphadenectomy for all mediastinal and abdominal regions, including the bilateral recurrent laryngeal nerve lymph nodes, should be performed in patients with esophageal squamous-cell carcinoma, whether in the early stage or having received preoperative chemoradiotherapy.

## Conclusion

Lymph node metastasis in esophageal squamous-cell carcinoma has a high incidence, occurs in the early stages, and is widely distributed in all regions of the mediastinum and abdomen. Minimally invasive esophagectomy and extended two-field lymph node dissection are feasible and safe, with a low complication rate.

## Ethical approval

This study was approved 4 April 2023 by Ethics Committee at Hanoi Medical University no: 875/GCN-HĐĐĐNCYSH-ĐHYHN.

## Consent

Written informed consent was obtained from the patient for the publication and accompanying images. A copy of the written consent is available for review by the Editor-in-Chief of this journal on request.

## Source of funding

Not applicable.

## Author contribution

D.D.N.: study design, data collection, data statistics, data interpretation, preparation of manuscript, literature analysis/search, resources, supervision. B.V.P.: data collection, investigation, supervision. M.D.T.: data collection. T.D.N.: data collection. A.D.T.: study design, data collection, data statistics, data interpretation, preparation of manuscript, literature analysis/search. K.V.L.: data collection. V.V.K.: investigation, supervision. H.X.N.: investigation, supervision.

## Conflicts of interest disclosure

The authors declare that they have no financial conflict of interest with regard to the content of this report.

## Research registration unique identifying number (UIN)

1. Name of the registry: researchregistry.com.
2. Unique Identifying number or registration ID: researchregistry10476.
3. Hyperlink to your specific registration (must be publicly accessible and will be checked): https://www.researchregistry.com/register-now#home/registrationdetails/668eb1df8010c10029116e21/.

## Guarantor

Duy Duc Nguyen, An Duc Thai and Binh Van Pham accept full responsibility for the work and the conduct of the study, had access to the data, and controlled the decision to publish.

## Data availability statement

Data are available upon reasonable request.

## Provenance and peer review

Not commissioned, externally peer-reviewed.

## Supplementary Material
